# QuickStats

**Published:** 2014-08-08

**Authors:** FROM THE NATIONAL CENTER FOR HEALTH STATISTICS

**Figure:**
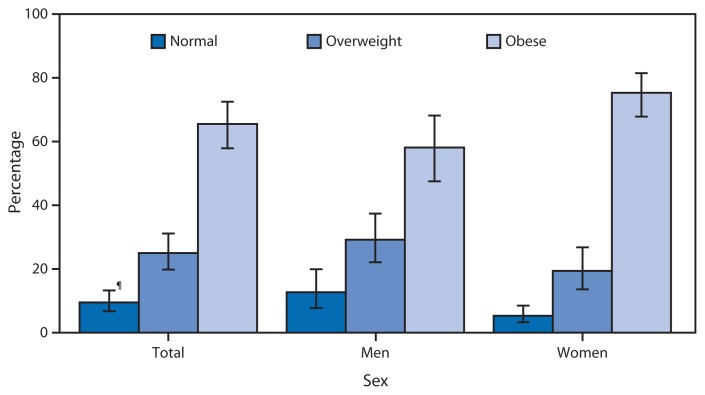
Percentage Distribution of Weight Status* Among Adults Aged ≥20 Years with Diabetes,^†^ by Sex — National Health and Nutrition Examination Survey, United States, 2009–2012^§^ * Weight status is based on body mass index cutoff values for adults (kg/m2): normal weight (18.5–24.9), overweight (25.0–29.9), and obese (≥30.0). ^†^ Diabetes is defined as a fasting plasma blood glucose ≥126 mg/dL, a hemoglobin A1c ≥6.5%, or a self-reported physician diagnosis of diabetes. ^§^ Estimates are age-adjusted to year 2000 U.S. Census standard population using age groups 20–39 years, 40–59 years, and ≥60 years. ^¶^ 95% confidence interval.

During 2009–2012, an estimated 65.5% of adults with diabetes were obese, 25.0% were overweight, and 9.5% were normal weight. The prevalence of obesity among women with diabetes (75.3%) was higher than the prevalence of obesity among men with diabetes (58.1%).

**Source:** CDC. National Health and Nutrition Examination Survey Data. Hyattsville, MD: US Department of Health and Human Services, CDC, National Center for Health Statistics; 2009–2012. Available at http://www.cdc.gov/nchs/nhanes.htm.

**Reported by:** Cheryl D. Fryar, MSPH, clf9@cdc.gov, 301-458-4537; Steven M. Frenk, PhD.

